# In search of epi-driver genes in head and neck cancer

**DOI:** 10.1186/1755-8166-7-S1-I23

**Published:** 2014-01-21

**Authors:** Sumana Bhattacharjya, Kumar Singha Roy, Nitai P Bhattacharyya, Susanta Roychoudhury

**Affiliations:** 1 Cancer Biology and Inflammatory Disorder Division, CSIR-Indian Institute of Chemical Biology, Kolkata, India; 2 Crystallography and Molecular Biology Division, Saha Institute of Nuclear Physics, Kolkata, India

## 

Over the last decade, sequencing of a large number of tumour genomes has identified thousands of mutations in many genes. Among these mutations that confer selective growth advantage to the tumour cell are called ‘Mut-driver’ mutations. Furthermore, it has been proposed that ‘Epi-drivers’ are a class of driver genes that are not frequently mutated but aberrantly-expressed in tumours through epigenetic means. Intriguingly, it has been stated that the most obvious source of the proverbial ‘dark matter’ is in Epi-driver genes and human tumours contain large numbers of epigenetic changes affecting DNA or chromatin proteins. However, it is now clear that microRNAs (miRNAs) also have specific epigenetic functions whereby they recognize and bind to specific mRNA targets to repress their expressions. Using head and neck cancer tumour model we are trying to identify such miRNAs and their target Epi-driver genes important in this cancer. In this quest, we have carried out an *in silico* investigation of 53 miRNAs known to be deregulated in head and neck squamous cell carcinoma (HNSCC) and the expression and mutation status of their experimentally-validated target genes in the disease. Interestingly, our results have put forward 224 target genes as potential Epi-drivers specific to HNSCC. How miRNA regulation of mitotic genes could contribute to the HNSCC development and thus might be considered as potential Epi-drivers, will be discussed.

